# Börjeson–Forssman–Lehmann syndrome: delineating the clinical and allelic spectrum in 14 new families

**DOI:** 10.1038/s41431-023-01447-0

**Published:** 2023-09-14

**Authors:** Vani Jain, Seow Hoong Foo, Stephen Chooi, Celia Moss, Richard Goodwin, Siren Berland, Angus J. Clarke, Sally J. Davies, Sian Corrin, Oliver Murch, Samantha Doyle, Gail E. Graham, Lynn Greenhalgh, Susan E. Holder, Diana Johnson, Ajith Kumar, Roger L. Ladda, Susan Sell, Amber Begtrup, Sally A. Lynch, Emma McCann, Rune Østern, Caroline Pottinger, Miranda Splitt, Andrew E. Fry

**Affiliations:** 1 All Wales Medical Genomics Service, University Hospital of Wales, Heath Park, Cardiff, CF14 4XW UK; 2 Department of Dermatology, Birmingham Women’s and Children’s NHS Foundation Trust, Birmingham, B4 6NH UK; 3 School of Medicine, Cardiff University, Heath Park Campus, Cardiff, CF14 4YS UK; 4 University of Birmingham, Edgbaston, Birmingham, B15 2TT UK; 5 Department of Dermatology, Royal Gwent Hospital, Newport, NP20 2UB UK; 6 Department of Medical Genetics, Haukeland University Hospital, 5021 Bergen, Norway; 7 Division of Cancer and Genetics, Cardiff University, Cardiff, CF14 4XN UK; 8 Department of Medical Genetics, Our Lady’s Children’s Hospital, Crumlin, Dublin, D12 N512 Ireland; 9 Department of Genetics, Children’s Hospital of Eastern Ontario, Ottawa, Ontario K1H 8L1 Canada; 10 Liverpool Centre for Genomic Medicine, Liverpool Women’s Hospital, Liverpool, L8 7SS UK; 11 North West Thames Regional Genetic Service, Kennedy Galton Centre, Northwick Park Hospital, Harrow, HA1 3UJ UK; 12 Department of Clinical Genetics, Northern General Hospital, Sheffield, S5 7AU UK; 13 North East Thames Regional Genetics Service, Great Ormond Street Hospital, London, WC1N 3JH UK; 14 Department of Pediatrics, Division of Human Genetics, Penn State Health Children’s Hospital, Hershey, Pennsylvania 17033 USA; 15 GeneDx, Gaithersburg, Maryland 20877 USA; 16 Department of Medical Genetics, St. Olavs Hospital, Trondheim University Hospital, 7030 Trondheim, Norway; 17 Northern Genetics Service, Newcastle upon Tyne Hospitals NHS Foundation Trust, Newcastle upon Tyne, NE1 3BZ UK; 18 Present Address: Department of Dermatology, Gleneagles Hospital Medini, Nusajaya, 79250 Johor Malaysia; 19 Present Address: Department of Clinical Genetics, The National Maternity Hospital, Holles Street, Dublin, D02 YH21 Ireland

**Keywords:** Disease genetics, Disease genetics, Genetic testing

## Abstract

Börjeson-Forssman-Lehmann syndrome (BFLS) is an X-linked intellectual disability syndrome caused by variants in the *PHF6* gene. We ascertained 19 individuals from 15 families with likely pathogenic or pathogenic *PHF6* variants (11 males and 8 females). One family had previously been reported. Six variants were novel. We analysed the clinical and genetic findings in our series and compared them with reported BFLS patients. Affected males had classic features of BFLS including intellectual disability, distinctive facies, large ears, gynaecomastia, hypogonadism and truncal obesity. Carrier female relatives of affected males were unaffected or had only mild symptoms. The phenotype of affected females with de novo variants overlapped with the males but included linear skin hyperpigmentation and a higher frequency of dental, retinal and cortical brain anomalies. Complications observed in our series included keloid scarring, digital fibromas, absent vaginal orifice, neuropathy, umbilical hernias, and talipes. Our analysis highlighted sex-specific differences in *PHF6* variant types and locations. Affected males often have missense variants or small in-frame deletions while affected females tend to have truncating variants or large deletions/duplications. Missense variants were found in a minority of affected females and clustered in the highly constrained PHD2 domain of PHF6. We propose recommendations for the evaluation and management of BFLS patients. These results further delineate and extend the genetic and phenotypic spectrum of BFLS.

## Introduction

Börjeson–Forssman–Lehmann syndrome (BFLS, MIM# 301900) is a rare form of syndromic intellectual disability (ID) first described in 1962 [1]. Pathogenic variants in the *PHF6* gene were identified as the cause of BFLS in 2002 [2]. BFLS in males is characterised by developmental delay, ID, obesity, gynaecomastia, hypogonadism, and dysmorphic facial features [2–4]. Carrier female relatives of affected males are usually unaffected or manifest only mild cognitive and physical features [3, 5, 6]. However, a growing number of female probands with de novo *PHF6* variants and severe symptoms have been described [7–15]. Here, we use the term ‘affected female’ to refer to this latter group.

The X-linked plant homeodomain finger protein 6 (*PHF6*) gene is highly conserved across vertebrates and intolerant to loss of function variants (gnomAD pLI = 1) [16, 17]. The PHF6 protein contains two PHD-like zinc finger domains (PHD1 and PHD2), two nuclear localisation signals, and a nucleolar localisation sequence [2]. PHF6 is an epigenetic transcriptional regulator implicated in neurogenesis and hematopoiesis [16, 18, 19]. PHF6 is highly expressed in the developing central nervous system [20]. Postnatally, PHF6 is ubiquitously expressed with high or moderate levels in the thymus, gonads, thyroid, spleen, adipose tissue and skin [21, 22].

Here, we describe the clinical and molecular findings in 19 individuals with likely pathogenic or pathogenic *PHF6* variants (17 new and updated details about two males previously reported as children) [23]. We compared our series with BFLS patients reported in the literature.

## Subjects and methods

Nine probands (M2, M3, M5–M8, F4, F7 and F8) had whole exome sequencing (WES) as part of the Deciphering Developmental Disorders (DDD) study [24]. Two additional males from family A (M1 and M4) had Sanger sequencing for the familial variant. The two previously reported males (family F, M9 and M10) had single gene *PHF6* testing [23, 25]. The rest of the series were tested by array comparative genomic hybridisation (aCGH) and/or WES. All research results were confirmed in a clinical laboratory.

Variant positions are based on *PHF6* transcript NM_032458.3. Variants were classified using guidelines from the American College of Medical Genetics and Genomics and Association for Molecular Pathology and the Association for Clinical Genomic Science [26, 27]. DECIPHER numbers and criteria used for variant classification are listed in Supplementary Table S1. Coding variants were assessed using a range of in silico prediction programs (Supplementary Table S2) [28].

A phenotyping questionnaire was devised based on the literature. Broad subheadings in the questionnaire included growth, development, learning, vision and hearing, neurobehavioral, skeletal, dental, cardiac, gastrointestinal, immune, dermatological, and other features. Detailed phenotyping was undertaken by a clinician responsible for the patient’s care. All individuals or their parents/guardians gave consent for publication.

## Results

We initially ascertained 20 individuals from 16 families (12 male, 8 female) with hemizygous or heterozygous variants in *PHF6* (Table 1). Detailed clinical descriptions are provided in the Supplementary material and Supplementary Table S3. Four males (M1–M4) were from family A. We also collected updated information from two brothers (M9 and M10, family F) who were previously reported [23, 25]. Pedigree diagrams for families A and F are shown in Supplementary Fig. S1. All other individuals were unrelated. The age range of the males was 2–34 years old. The females were 8–26 years old.

**Table 1:** Individuals with *PHF6* variants reported in this study.

Individual	Sex	Age	PHF6 variant (NM_032458.3)	Inheritance	Classification
M1 (family A)	Male	13 y	c.1014_1016del, p.(Glu338del)	Maternal	P
M2 (family A)	Male	27 y	c.1014_1016del, p.(Glu338del)	Maternal	P
M3 (family A)	Male	5 y	c.1014_1016del, p.(Glu338del)	Maternal	P
M4 (family A)	Male	6 y 3 m	c.1014_1016del, p.(Glu338del)	Maternal	P
M5 (family B)	Male	7 y 5 m	c.1014_1016del, p.(Glu338del)	Maternal	P
M6 (family C)	Male	8 y	c.1057G>C, p.(Asp353His)	Maternal	VUS
M7 (family D)	Male	2 y 5 m	c.1024C>T, p.(Arg342*)	De novo	P
M8 (family E)	Male	7 y	c.686A>G, p.(His229Arg)	Maternal	P
M9 (family F)	Male	20 y	c.139-8A>G, p.(?)	Maternal	P
M10 (family F)	Male	13 y	c.139-8A>G, p.(?)	Maternal	P
M11 (family G)	Male	34 y	c.2T>C, p.(Met1?)	Unknown	P
M12 (family H)	Male	10 y 2 m	c.146C>T, p.(Ser49Leu)	Maternal^a^	LP
F1 (family I)	Female	15 y	c.743G>T, p.(Gly248Val)	De novo	P
F2 (family J)	Female	26 y	206 kb deletion (exons 6 to 10)	De novo	P
F3 (family K)	Female	11 y	11 kb deletion (exons 4 and 5)	De novo	P
F4 (family L)	Female	12.5 y	c.955C>T, p.(Arg319*)	De novo	P
F5 (family M)	Female	9 y	Whole gene deletion	De novo	P
F6 (family N)	Female	10 y	c.732G>C, p.(Leu244Phe)	Not maternal	LP
F7 (family O)	Female	15 y	c.719A>G, p.(Tyr240Cys)	De novo	P
F8 (family P)	Female	8 y 9 m	c.297T>A, p.(Cys99*)	De novo	P

Key: Age, at last review in y(ears) and m(onths); ACMG classification, P(athogenic), LP (likely pathogenic) and VUS (variant of uncertain clinical significance).

^a^The p.(Ser49Leu) variant was de novo in the mother.

Ten affected males inherited their *PHF6* variants from carrier mothers. The mothers were unaffected or had only mild symptoms. The recurrent p.(Arg342*) variant detected in individual M7 was de novo. The carrier status of one mother was unknown. A small in-frame deletion, p.(Glu338del), was found in the four affected males of family A and another apparently unrelated male (M5). The other males had missense, start loss, or splice site variants. Three variants in the males were novel, p.(Glu338del), p.(Asp353His) and p.(Ser49Leu). Seven affected females were confirmed to have de novo *PHF6* variants. The father of individual F6 was not available for testing but maternal testing was negative. Four females had novel variants. These included one nonsense variant (p.(Cys99*)) and three novel missense variants (p.(Tyr240Cys), p.(Leu244Phe) and p.(Gly248Val)). The missense variant in individual M6 (p.(Asp353His)) was classified as a variant of uncertain clinical significance (the variant was not in gnomAD but in silico predictions were mixed and photographs of M6 were not available to review). The details of M6 were therefore excluded from our subsequent analysis. All other variants were classified as pathogenic or likely pathogenic (Table 1).

### Pregnancy, perinatal period and infancy

There were few complications during pregnancy. One male and one female were born prematurely. Birth weight was in the normal range with no reports of intrauterine growth restriction. Hypotonia and feeding difficulties were common in infancy. Congenital anomalies reported in the series included umbilical hernias (1 male/4 females), talipes (2 females) and structural kidney anomalies (2 males/1 female).

### Growth

Many individuals were described as obese, had truncal obesity, or a BMI > 2 standard deviations (SD) above mean for age (9/10 males, 6/7 females). Short stature was common. Height was below average in 9/10 males and more than 2 SD below the mean in 4/10. Height in affected females ranged from −2.7 SD in individual F3 to +1.8 SD in individual F6, but other females (5/7) were within 1 SD of mean height for age. Occipitofrontal circumference (OFC) was also variable. One male (M2) and two females (F6 and F7) had macrocephaly (OFC > +2 SD). In contrast, one male (M7) was microcephalic (OFC −2.5 SD). Three other males and three females had relatively small heads (OFC −1 to −2 SD).

### Physical features

**Fig. 1:**
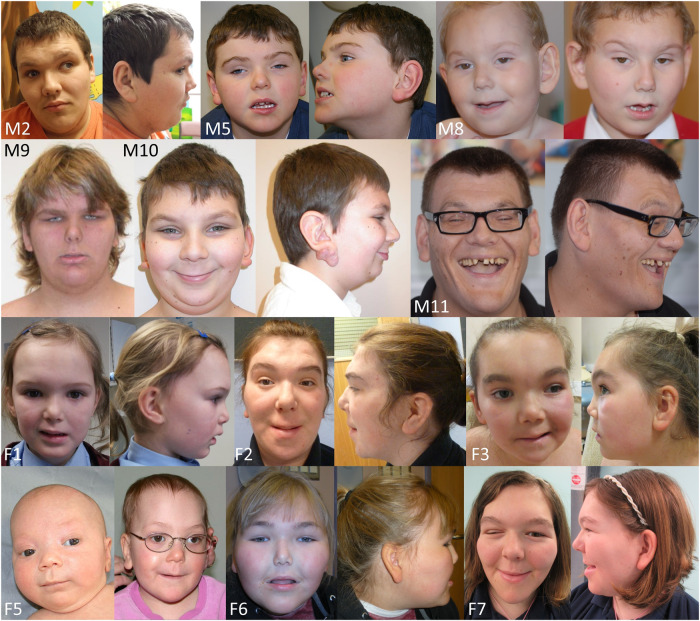
Facial phenotype of males and females with BFLS. Photographs show individual M2 at age 26 years; individual M5 at age 6.5 years; individual M8; individual M9 at age 17 years; individual M10 at age 11 years; individual M11; individual F1 at age 13 years; individual F2 at 16 years of age; individual F3 at age 8 years; individual F5 at age 2 months and 3.5 years; individual F6 at age 9 years; and individual F7 at age 15 years. The dysmorphic facial features in both sexes include deep-set eyes, narrow palpebral fissures, large fleshy ears lobes, and short noses with a bulbous nasal tip. Facial features tend to coarsen with age.

Photographs of 12 individuals are shown in Fig. 1. Typical facial features of BFLS were present in males and females. These included deep-set eyes, up-slanting palpebral fissures, hypertelorism, ptosis, long ears with fleshy ear lobes, and thin upper lip. Facial features tended to coarsen with age leading to a prominent brow and bulbous nose. Males and females had short, tapering fingers often with 5th finger clinodactyly and broad feet with short toes (Supplementary Fig. S2). Additional digital anomalies noted in females included toe syndactyly (5 females) and hypoplastic or dysplastic toe/fingernails (5 females).

### Neurodevelopment, behaviour and neurology

All the individuals had delayed motor milestones. Intellectual disability varied in severity from mild to severe. Limited or delayed speech was common (18/19). Behavioural problems were also frequent (13/19, 6 males and 7 females). These included autistic traits, food seeking behaviour or hyperphagia, and challenging or aggressive behaviour. Three females had repetitive complex motor behaviours (hand wringing, tics or stereotypies).

Individual F3 in our series had a distal lower limb motor axonal neuropathy. Two males and one female had a history of seizures. In addition, individual F6 had a history of staring episodes but no formal diagnosis of epilepsy. Nine individuals (4 males and 5 females) had had MRI brain scans. Five were abnormal. Individual F1 had an area of cortical dysplasia involving blurring of grey-white matter junction in the right frontal lobe extending into the right insula and possibly the right temporal lobe. Individual F5 had increased signal in the periventricular white matter and a relatively large cerebellum, individual F8 had hypoplasia of the corpus callosum and cavum septum pellucidum, and two males had reduced (M5) or delayed myelination (M8).

### Hearing and vision

Nine individuals had a history of recurrent otitis media or conductive hearing loss or had required grommet insertion. Two had mild sensorineural hearing loss. Visual problems were common and included refractive errors (6 males and 5 females), strabismus (2 males and 5 females) and nystagmus (M3 and M8). Individual F2 had atrophic pigmentary changes of the retina and choroid. Individual F4 had dysplastic optic discs.

### Skin, hair, nails and teeth

**Fig. 2:**
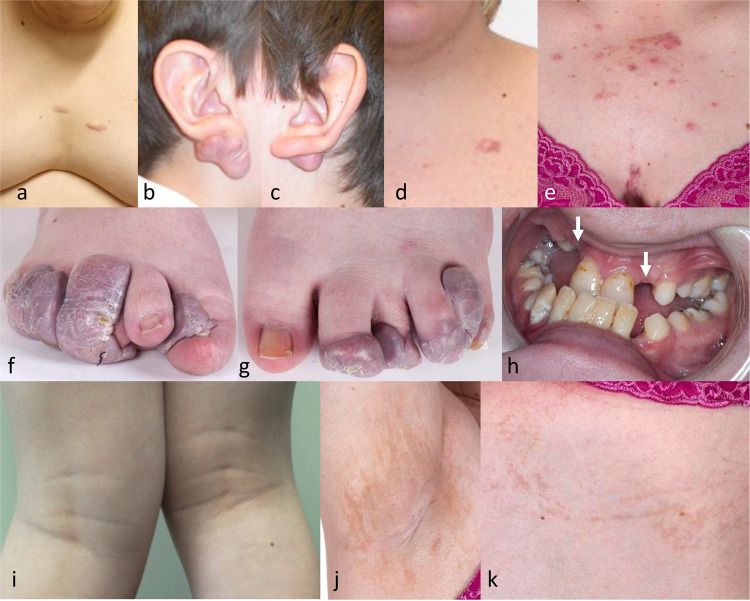
Skin and dental features of patients with BFLS. Hypertrophic or keloid scarring on the chest of individual M2 (**a**), the ears of individual M10 (**b**,**c**), chest of individual M9 (**d**) and chest of individual F2 (**e**). Individual F2 had large fibromas grow from the nail beds of 8 toes (**f**, **g**). Dental examination of individual F2 (**h**) found a hypoplastic maxilla leading to a prognathic mandible and a range of dental anomalies including hypodontia, small widely spaced and irregularly shaped teeth, double talon cusps, dens invaginatus, and enamel hypoplasia. Teeth were removed due to compound composite odontomas (arrows). Steaky skin hyperpigmentation seen on individuals F1 (**i**) and F2 (**j**, **k**).

Hypertrophic or keloid scarring was observed in four individuals (3 males and 1 female) (Fig. 2a–e). A BFLS patient with keloids has previously been reported by Turner et al. [3]. Individual M2 in our series developed keloids on his chest and arms due to skin picking. M9 had a single keloid on his chest. M10 (younger brother of M9) developed significant keloids after otoplasty. Individual F2 had multiple keloids across her chest (from acne) and limbs (from scratching). Starting from 9 years of age, F2 also developed large bluish fibromas from the nail beds of eight toes (Fig. 2f,g). The initial trigger for these is uncertain but her toenails were noted to be small, deep-set and difficult to cut. The fibromas caused significant distress from pain and discharge and regrew after surgical attempts to clear them. Clinically and histologically, they resembled the inclusion body-negative digital fibromas seen in terminal osseous dysplasia with pigment defects (TODPD, MIM# 300244). At least six affected females were noted to have streaky or swirly patches of hyperpigmentation following Blaschko’s lines (Fig. 2i–k). Individual M12 had several hypopigmented macules and café au lait patches. Individual M10 also had multiple pigmented naevi. Sparse, fine or slow growing hair was reported in four females and two males. Nails tended to be small and horizontally curved, corresponding to the tapering shape of fingers, or shortened due to terminal brachydactyly. Abnormal dentition was common in females (6/8) but also reported in a minority of males (3/11).

### Endocrinology

Small external genitalia or features of hypogonadism were noted in six males and four females. Undescended testes were present in three. Individual M1 had delayed puberty requiring testosterone supplements. Two males (M2 and M8) had growth hormone deficiency. Gynaecomastia was reported in 5 males (predominantly the older males, aged 10–34 years). Three females (F1, F5 and F7) had primary amenorrhoea. Individual F1 had absent ovaries and only a rudimentary uterus. In contrast, individual F2 had progressed through puberty normally. The four other females in our series were still pre-pubescent although individual F4 had hypoplastic labia and no vaginal orifice. Affected females with hypoplastic labia have previously been reported [9–11].

## Discussion

We present the clinical and molecular details of 19 individuals with pathogenic or likely pathogenic *PHF6* variants. This adds to the approximately 90 molecularly confirmed BFLS patients described in the literature [15]. We compared our series with a review of previous reports compiled by Gerber et al. (Table 2) [15]. There was a strong overlap between the male and female BFLS phenotype. Shared clinical features include early developmental delay, intellectual disability and obesity. Characteristic dysmorphism in both sexes include deep-set eyes, narrow palpebral fissures, large, fleshy ears, bulbous nasal tip, tapering fingers, and broad feet with short and/or flexed toes. Facial features tend to coarsen with age beginning in late childhood in association with the onset of obesity [3, 23]. Relatively few individuals in our series were reported to have hypogonadism (4/9 males and 4/8 females compared with rates close to 100% in previous patients). This may reflect under ascertainment due to the young age of our series.

**Table 2:** Comparison between our series and previous BFLS patients.

	Current Study	Published	Current Study	Published
	*n* = 9^a^	*n* = 62^b^	*n* = 8	*n* = 27^b^
	Males	Males	Females	Females
De novo variant	1/8	1/56	7/7	22/26
Height	4 short, 4 normal	9 short, 12 normal	1 short, 6 normal	2 short, 17 normal, 4 tall
Head size	1 micro-, 5 normo-, 1 macrocephaly	5 micro-, 25 normo-, 6 macrocephaly	6 normo-, 2 macrocephaly	4 micro-, 14 normo-, 2 macrocephaly
Obesity	7/8	44/48	6/7	5/25
Intellectual disability	9/9	54/54	8/8	24/27
Seizures	2/9	x3	1/8	5/23
MRI anomalies	2/4	NR	3/5	10/15
Behavioural anomalies	4/9	13/34	6/8	6/15
Finger anomalies	7/9	16/16	7/8	24/25
Toe anomalies	6/9	21/22	7/8	21/23
Linear skin hyper-pigmentation	NR	NR	7/8	17/25
Dental anomalies	2/9	1× small teeth, widely spaced	6/8	18/20
Hypogonadism or small external genitalia	4/9	44/45	4/8	10/10
Eye anomalies	8/9	3/5	7/8	13/15

*NR* not reported.

^a^Individuals M9 and M10 were not included in this column as they were previously reported.

^b^Numbers for previous patients are taken from ref. [15].

Several key differences between the male and female BFLS phenotype were noted. Affected males were typically short while females were often of average stature with occasional short or tall individuals. Gynaecomastia was a distinctive feature in older males. Streaky hyperpigmentation in a Blaschkoid distribution, consistent with X-chromosome inactivation (XCI), was a distinctive feature of affected females [8, 10, 11]. Affected females had a high rate of dental anomalies (6/8 in our series, 18/20 in previous patients [15]) compared to only 3 males across the combined dataset. Digital anomalies were common in both males (combined dataset 23/25, 92%) and females (combined dataset 31/33, 94%). In our series the digital anomalies in males tended to be relatively mild (e.g., short, tapering fingers). In contrast, the digital anomalies in females included clinodactyly of the 4th and 5th fingers, brachytelephalangy and hypoplastic nails. Similarly, although refractive errors and strabismus are common in both affected males and females, it is the latter who are particularly noted to have retinal abnormalities including retinal dystrophy [10], retinal depigmentation with maculopathy [15], and bilateral optic nerve dysplasia and synovial hypoplasia of the retina [29]. Individual F2 in our series had atrophic pigmentary changes of the retina and choroid, and individual F4 had dysplastic optic discs. A summary of common clinical features in male and female BFLS patients is given in Table 3.

**Table 3:** Summary of common clinical features in male and female BFLS patients.

Common features	**Early Life**	
	Hypotonia	Feeding difficulties
	Delayed motor milestones	Failure to thrive
	**Childhood**	
	*Facial features*	*Other features*
	Prominent supraorbital ridges	Delayed speech
	Deep set eyes	Intellectual disability
	Narrow, upslanting palpebral fissures	Behaviour problems
	Hypertelorism	Obesity
	Ptosis	Recurrent otitis media
	Thin upper lip	Hearing loss
	Bulbous nasal tip	Refractive errors
	Coarsening facial features	Broad feet with short toes
	Long ears with large, fleshy earlobes	Abnormal dentition
	**Sex-specific**	
	*Males*	*Females*
	Short stature	Linear skin hyperpigmentation
	Gynaecomastia	Clinodactyly/syndactyly
	Short, tapering fingers	Hypoplasia/dysplasia of the nails
	Undescended testes	Primary amenorrhoea
	Small genitalia	Genital anomalies
		Sparse hair
		Cortical brain anomalies
Less common features	Endocrine: growth hormone deficiency, hypogonadism, hypothyroidism, pituitary anomalies; seizures; congenital anomalies: talipes, umbilical hernia and structural kidney anomalies; retinal and/or optic disc anomalies (females); repetitive complex motor behaviours; skin anomalies: hypopigmented patches, café au lait, naevi and hypertrophic/keloid scarring	

**Fig. 3:**
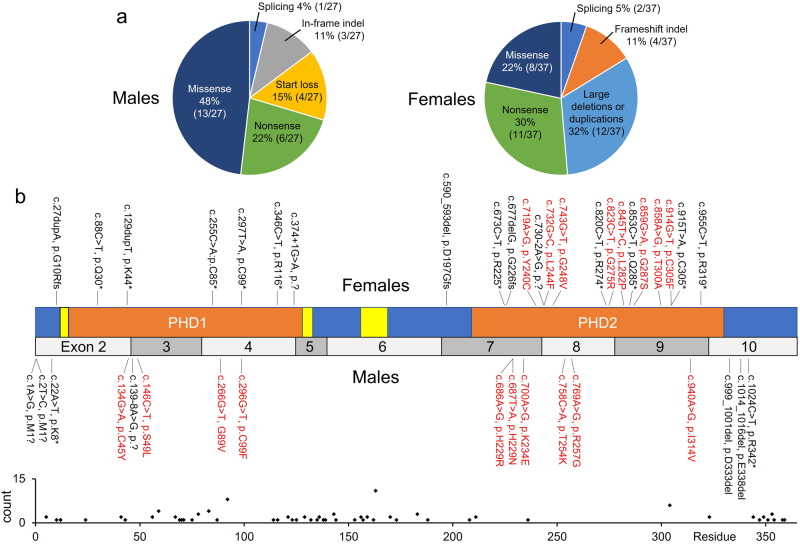
Comparison of *PHF6* variants found in affected males and females. **a** *PHF6* mutation types in families (our series plus literature) grouped by sex of BFLS probands. **b** Model of *PHF6* gene showing location of BFLS associated sequence variants. The domain structure of the PHF6 protein is shown with exons below. The PHD-like domains (orange rectangles) containing zinc fingers are PHD1 (residues 14–132) and PHD2 (residues 209-330). PHF6 contains two nuclear localisation sequences (yellow boxes, residues 13–16 and 129–133) and a nucleolar localisation sequence (yellow box, residues 157–169). Domains positions are based on UniProtKB Q8IWS0. Exon model based on transcript NM_032458. *PHF6* variants reported in affected females and males are shown above and below the model respectively. Missense variants are in red text. Large deletions and duplications are not shown. Below the model are plotted allele counts for missense variants from gnomAD (v2.1.1) demonstrating the severe depletion of variation across the PHD2 domain in the general population.

To study the difference in variant types between affected males and females, we analysed the types and locations of *PHF6* variants in our series combined with the literature (Fig. 3, Supplementary Table S4). No pathogenic variant has yet been reported in both an affected male and de novo in an affected female. A female in our series had a nonsense variant at codon 99, the same residue as a missense previously reported in an affected male [2]. The variant types observed in males included missense variants, start loss, small in-frame deletions, splice site, and nonsense variants (Fig. 3a). Missense variants and small in-frame deletions are frequently reported in males (59%, 16/27 families) and are distributed across the gene, including both PHD domains. The recurrent p.(Arg342*) variant has now been observed on at least five occasions including the original BFLS family [30, 31]. Truncating (*n* = 7) and start loss variants (*n* = 4) in males have so far been confined to exons 2 and 10 (the first and last coding exons) raising the possibility that transcripts might use alternative initiation codons or escape nonsense mediated decay, producing truncated (rather than absent) proteins resulting in milder clinical effects. No males with large copy number variants involving multiple exons or the whole gene have been observed. In contrast, numerous large duplications and deletions have been observed de novo in affected females (Fig. 3a, 32%, 12/37) [11, 15]. Nonsense and frameshift variants are common in affected females (46%, 17/37) and are distributed across the gene (Fig. 3b). Missense variants are less common in affected females (22%, 8/37; including the three novel missense variants found in our series) and cluster in the PHD2 domain. The PHD2 domain is highly constrained in the general population with little gnomAD variation across the region (Fig. 3b). The clustering of pathogenic missense variants in BFLS females in combination with the population variant data is compelling evidence for the functional significance of the PHD2 domain.

Our findings support the observation that de novo variants occurring in affected females tend to be more severe and result in loss of PHF6 while inherited variants identified in males are more likely to result in residual PHF6 activity due to milder effects on protein stability or function [32]. A similar situation exists for other X-linked genes such as *IKBKG* (loss-of-function variants cause incontinentia pigmenti (MIM# 308300) in females; variants with less severe consequences cause hypohidrotic ectodermal dysplasia and immunodeficiency (MIM# 300291) in males) [33]. This could suggest that hemizygous males with severe loss-of-function *PHF6* variants may not be viable; therefore, only males with milder *PHF6* variants are ascertained. In contrast, females with severe loss-of-function *PHF6* variants survive but manifest disease, while females with milder alleles have low penetrance (or mild expression) and may only be ascertained if they have an affected child. Another potential explanation why some heterozygous females are severely affected may be differences in XCI. Severely affected females (including F1 and F2 in our series) have highly skewed XCI in blood [8, 10, 15]. However, so do some healthy carrier females in BFLS families, while others do not [2, 3, 5, 7]. Furthermore, in two cases of mother-to-daughter transmission of truncating *PHF6* variants, similar levels of skewing were seen in the mildly affected mothers and more severely affected daughters [15, 34]. Therefore, a clear correlation between XCI and disease severity has not been demonstrated. However, most XCI testing to date has been of blood leucocytes which may not reflect the status in key tissues.

Epilepsy was reported in two males from the original BFLS family but only occasionally in subsequent BFLS males [1, 3, 35, 36]. Two males in our series had a history of seizures (possibly due to recurrent hypoglycaemia in one). The frequency of epilepsy in affected females may be higher with 5/23 reported in the literature [15]. An affected female (F1) with medically resistant frontal epilepsy was observed in our series. Affected females often have structural brain abnormalities, including 10/15 in the literature and three in our series [15]. Malformations of cortical development (MCD) are often reported. Two previous affected females with intragenic duplications in *PHF6* had simplified gyration with subcortical band heterotopia in the temporal and peri-insular regions [14, 15]. Blurred grey-white demarcation was seen in their frontal lobes. Individual F1 in our series had similar blurring of the grey-white matter in the right frontal, temporal, and insular regions. Possible blurring of the grey-white boundary and frontal subcortical heterotopia have been observed in other affected females [14, 15]. PHF6 has been implicated in the regulation of neurogenesis and neuronal migration through interactions with factors such as PAF1, NuRD and miR-128 [37–39]. RNAi knockdown of PHF6 in mouse cerebral cortex severely impairs neuronal migration causing band-like heterotopic aggregates which display hyperexcitability [37]. Furthermore, knockout of PHF6 in human neuron-like cells demonstrated impaired neurite outgrowth, proliferation and migration [32].

In addition to MCDs, white matter abnormalities are also reported in BFLS. The two females with MCDs reported by Kasper et al. also had periventricular white matter hyperintensities [14]. Individual F5 in our series had increased signal in the periventricular white matter and individual F7 had a hypoplastic corpus callosum. Non-specific increase in white matter signal sometimes with enlarged ventricles have been reported in other affected females [8, 15, 34]. Reports of brain scan findings in BFLS males are limited. Two males in our series had absent or delayed myelination. A previously reported affected male had mild corpus callosum hypoplasia while his brother had atrophy of the posterior corpus callosum and an abnormal pituitary gland [35]. The impact of BFLS on neurons may extend outside of the central nervous system. Axonal neuropathy was observed in individual F3 in our series. Polyneuropathy has previously been reported in a BFLS carrier mother, her affected sons and brother [31].

The phenotypic combination of ID, sparse hair and hypoplastic nails in younger affected females has been noted to overlap with Coffin-Siris syndrome (MIM# 135900) [11]. This was a diagnosis considered for individual F4. However, the ectodermal (skin, hair, teeth, brain and retinal) abnormalities in BFLS females are also reminiscent of other X-linked dominant disorders. The streaky skin pigmentation in F1, F3 and F5 led to incontinentia pigmenti being considered and *IKBKG* gene testing being arranged. Similarly, a diagnosis of TODPD was considered for F2 due to her combination of pigmentary abnormalities, digital anomalies, and recurrent digital fibromas. Furthermore, the grey matter heterotopia occasionally reported in BFLS females may be a manifestation of functional mosaicism in a manner similar to X-linked MCDs [40, 41].

A range of recommendations for the initial evaluation and management of BFLS patients are presented in Supplementary Tables S5 and S6. These highlight the importance of neurodevelopmental follow-up, endocrinology review, eye checks and skin care. At present, we have not suggested specific surveillance for cancer. Somatic *PHF6* mutations have been found in haematological cancer and may play a role in other cancer types [21, 30, 42–44]. Several germline BFLS variants have been reported as somatic mutations in cancer. These include p.(Gly10Argfs*) (in T-cell acute lymphoblastic leukaemia (T-ALL)), p.(Arg274*) (in T-ALL and acute myeloid leukaemia) and p.(Arg342*) (in T-ALL) [21, 42]. However, only three cancers have been reported in BFLS patients so far. Hodgkin Lymphoma and T-ALL have been reported in two BFLS males [23, 30]. Individual F8 in our series developed a Wilms tumour at the age of 3 years. Therefore, at present, the cancer risk for BFLS patients remains uncertain due to lack of evidence.

In summary, we have reported the clinical and molecular findings in 19 individuals (17 new) with pathogenic or likely pathogenic *PHF6* variants. We compared our series with previously reported individuals. The affected males in our series demonstrated classical features of BFLS including intellectual disability, distinctive facies, large ears, gynaecomastia, hypogonadism, and truncal obesity. We found the phenotype of affected females overlaps with males but includes streaky skin hyperpigmentation and a higher frequency of dental, retinal and cortical brain anomalies. Additional complications observed in our series included keloid scarring, digital fibromas, umbilical hernias, absent vaginal orifice, neuropathy and talipes. Our analysis highlights the differences in *PHF6* variant type and location between affected males and females, and the clustering of de novo missense variants in the PHD2 domain in affected females.

### Supplementary information

Supplementary tables, figures and case summaries

Supplementary Table 1

Supplementary Table 2

Supplementary Table 3

Supplementary Table 4

## Data Availability

The data that support the findings of this study are available from the corresponding author upon reasonable request. All sequence variants have been submitted to either the DECIPHER database (DECIPHER identifiers 273196, 295010, 294422, 280090, 274397, 259005, 258725, 304952, 286430) or ClinVar (Accession numbers VCV000011071, VCV001303761, SCV003925760, SCV003925761 and SCV003925762).
