# Supplementary material for: Börjeson–Forssman–Lehmann syndrome: delineating the clinical and allelic spectrum in 14 new families
Source: Eur J Hum Genet. 2023 Sep 14;31(12):1421–9. doi: 10.1038/s41431-023-01447-0 (PMC10689765; doi:10.1038/s41431-023-01447-0)
Supplement: Supplementary file 1 — Supplementary tables, figures and case summaries [file 41431_2023_1447_MOESM1_ESM.pdf]

Supplementary Table 5. Recommended evaluations following initial diagnosis in individuals with Börjeson-Forssman-Lehmann syndrome.

System/Concern	Evaluation	Comment
**Growth**	Plot height, weight, & head circumference on age-appropriate charts.	
**Development**	Developmental assessment; evaluation for occupational, speech, or physical therapy as needed	
**Eyes**	Ophthalmology examination and check of visual acuity	Consider dilated fundus examination in females
**Neurology**	Neurologic exam; assessment for seizures or neuropathy	MRI brain if seizures or focal neurology
**Hearing**	Audiology evaluation to assess for hearing loss	
**Education**	Educational assessment	
**Integument**	Clinical assessment for skin findings	
**Genetic counselling**	By medical geneticist or certified genetic counsellor	To inform patient and their family about inheritance pattern and implications of BFLS; to facilitate medical & personal decision making
**Kidneys**	Renal ultrasound to evaluate for structural kidney or genitourinary anomalies	
**Teeth**	Dental assessment	
**Endocrine**	Assess pubertal development; check levels of pituitary hormones including growth and sex hormones	Specialist endocrine review should be considered for all BFLS patients but particularly those with short stature, abnormal genitalia, features of hypogonadism or delayed puberty.

Supplementary Table 6. Prevention of secondary complications and treatment of manifestations in individuals with Börjeson-Forssman-Lehmann syndrome.

Manifestation/Concern	Evaluation/Treatment
**Obesity**	Early referral to dietician if food seeking or hyperphagia
**Hyperplastic scaring/keloids**	Avoid cosmetic surgical procedures; avoid injuring skin (e.g., body piercings); good wound care following essential surgery.
**Hypogonadism, primary/secondary amenorrhea**	Endocrinology review for adolescent BFLS patients
**Short stature**	Endocrinology review to consider growth hormone therapy
**Developmental delay / Intellectual disability**	Regular evaluation by a developmental paediatrician to assess developmental progress and educational interventions. Occupational, physical, and/or speech therapies to optimize developmental outcomes
**Seizures**	Management by neurologist with experience of treating epilepsy
**Hearing impairment**	Follow up of audiologic abnormalities if present. Hearing aids as needed; referral to otorhinolaryngologist if chronic otitis media
**Visual impairment**	Follow up of ophthalmologic abnormalities if present. Spectacles to correct refractive errors. Surgery if needed for strabismus and/or ptosis.

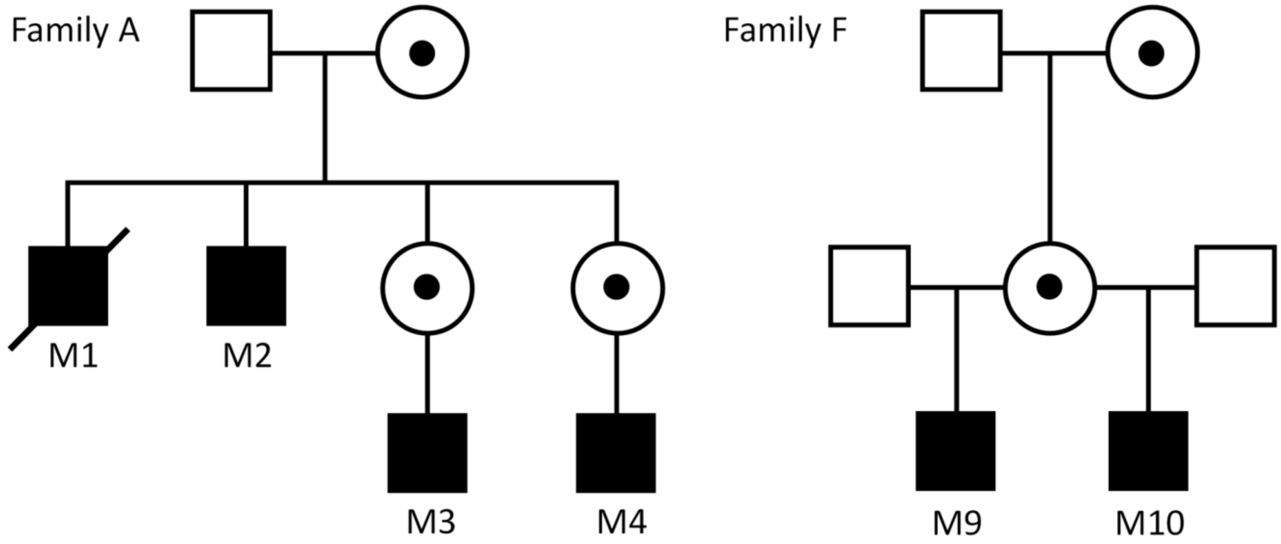

Supplementary Fig. 1. Pedigrees diagrams for families A and F. Males with Börjeson-Forssman-Lehmann syndrome (BFLS, black squares) and their carrier female relatives (circles with dots) are shown. Carrier females in these families were unaffected or manifested only mild features.

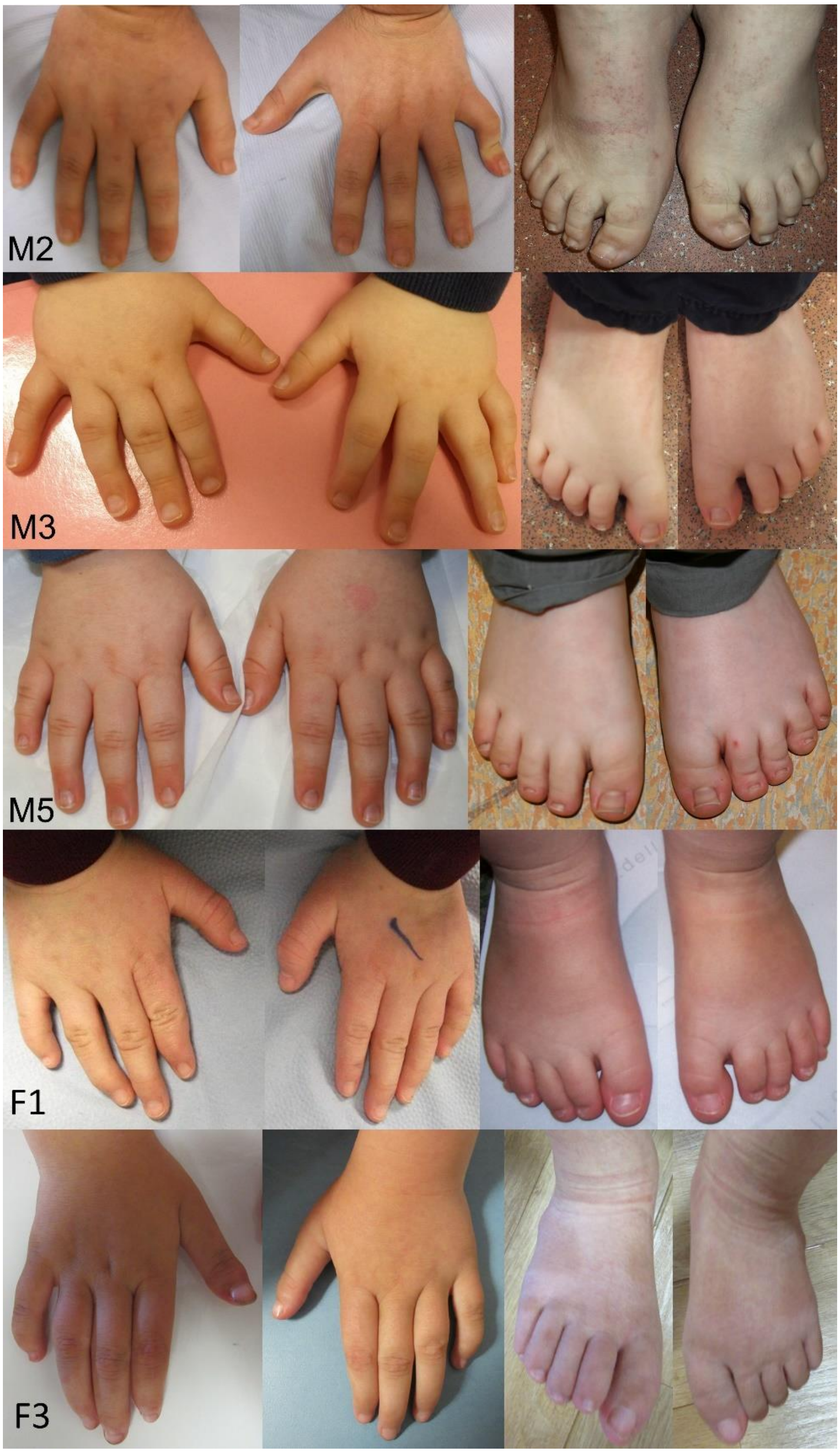

Supplementary Fig. 2. Hands and feet of individuals with BFLS. Hands and feet of individuals M2, M3, M5, F1 and F3 are shown. Note tapering fingers with brachyclinodactyly, particularly of fingers IV and V. The toes are short, broad and often clawed. Toenails are often short corresponding to terminal brachyphalangy. Some big toes nails are excessively curved horizontally.

Detailed case summaries

Family A, individual M1

This male patient was the brother of M2 and maternal uncle of individuals M3 and M4. He was born at term by normal vaginal delivery with a birth weight of 3033 g (-1.1 standard deviations (SD) below mean for age and sex). He had marked feeding problems, failure to thrive and hypotonia. He was noted to have a horseshoe kidney and undescended testes, for which he required surgery. He sat at 18 months and walked at 26 months of age. As a baby, he was frequently constipated. A rectal biopsy was normal. He had surgery for ileocolic intussusception at age 1 year. The patient's family recall that he sweated profusely as a baby. There were no documented episodes of hypoglycaemia. His speech was delayed. He began to put two words together from age 3 years.

At age 7 years, he was referred to Clinical Genetics, along with his younger brother (individual M2). At the time he was being educated in a special school. His family mentioned there had been a loss of some abilities as he had been able to name colours. He required insertion of grommets for glue ear. On examination, he had slightly coarse features with thick lips and up-slanting palpebral fissures. He was brachycephalic with a head circumference of 53cm (-0.3 SD). He remained hypotonic.

At age 13 years, he had marked learning difficulties and was very obese with a slight back hump. He had full lips, long ears (7.2 cm), small feet and small genitalia. His height was 127.3 cm (-3.5 SD). He was reported to have delayed puberty and required testosterone supplementation. He was known to be long-sighted.

As an adult, individual M1 had a placid and friendly temperament. He put on significant amounts of weight but in his 20's began to develop frequent infections of his chest, nose, eyes and ears. He lost a significant amount of weight. At age 26 years of age, he died of *H. Influenzae* pneumonia. Immune investigations were incomplete, but his working diagnosis was common variable immunodeficiency.

Investigations included urine glycosaminoglycans, CT head, growth hormone, skeletal survey, testing for Fragile X syndrome and a routine karyotype. Results were all normal. An EEG showed no specific abnormality. Further genetic investigations were carried out on his brother (individual M2) as their phenotypes were similar. Sanger sequencing on stored DNA confirmed individual M1 shared the familial *PHF6* c.1014_1016del, p.(Glu338del) variant.

Family A, individual M2

This male patient was the brother of M1 and maternal uncle of individual M3 and M4. He was born at 29 weeks gestation with a birth weight of 1750 g (+2.1 SD). He had an intraventricular haemorrhage and later developed right sided porencephalic cysts and periventricular leukomalacia. At 18 months old, he had no words and immature fine motor skills. His head circumference was 47.7cm (-1.2 SD) and he had large, low-set ears and up-slanting palpebral fissures. He sat at 15 months old and walked at 21 months and required grommets at a young age.

Individual M2 started having seizures in the first year of life. These continued until the age of 7 years. Endocrine investigation at 7 years of age recorded a low blood glucose, impaired growth hormone response and a low IGF-1 (5.2nmol/l; normal range 11.5 to 34.6nmol/l). His cortisol response and GHRH stimulation test were normal. A defect in the hypothalamic control of pituitary growth hormone release was considered. An MRI scan showed his pituitary stalk extended from the hypothalamus to the pituitary, but the posterior pituitary appeared small. At age 7 years, individual M2 began to eat more and gain weight. His head circumference was 52.5 cm (-0.7 SD) and height 103.5cm (-3.6 SD). He was long-sighted with a squint, 5th finger clinodactyly, full lips and full eyebrows. He had growth hormone therapy in childhood which stopped at 15 years of age due to poor compliance and lack of response.

At 16 years of age he had central obesity, acanthosis nigricans, and small hands and feet. He sucked his fingers excessively and appeared to have a high pain threshold. He frequently picked his skin till it bled. There were no problems with puberty.

At 27 years of age, individual M2 lived at home with his family and had carers that assisted with personal care. Due to difficult behaviour that was at times physically aggressive, he was under the care of an adult psychiatrist. Occasionally, his behaviour towards female family members was of a sexualised nature. On examination his weight was 116.8 kg (+3.2 SD), height 161.9cm (-2.4 SD) and OFC 61.5cm (+2.5 SD). He had deep set eyes, hypertelorism, a left sided squint, large fleshy ear lobes, bulbous nose with short philtrum and full lips. His palms and feet were broad with short fingers and toes. He had gynaecomastia, acanthosis nigricans, sparse body hair and rarely needed to shave. He had keloid scarring on his chest and arms due to skin picking. He had a normal LH, FSH and diabetic screen.

Individual M2's genetic investigations included tests for Prader-Willi syndrome, Fragile X syndrome, *MECP2*, *ARX*, routine karyotype and 1Mb array CGH. The results were all normal. An X-linked recessive condition was strongly suspected, and some family members had opted for prenatal fetal sexing. Individual M2 was recruited to the DDD study (DECIPHER 273196). However, after single gene testing in individual M3 identified a 3 base pair deletion in *PHF6* c.1014_1016del, p.(Glu338del), individual M2 was also tested and confirmed to share the same variant.

Family A, individual M3

Individual M3 was the maternal cousin of individual M4. His mother was the sister of individuals M1 and M2. He was born at 40+1 weeks gestation by emergency caesarean section due to face presentation. He was the couple's first child and scans during the pregnancy were normal. His birth weight was 3742 g (+0.4 SD) and his birth OFC was 38cm (+2.2 SD). He required syringe feeding as he struggled to breast feed and was losing weight. The feeding issues eventually resolved. At 4 months of age, the parents of individual M3 described him as "not doing much and very floppy". Head control was achieved at 4.5 months, crawling at 15 months and walking at 24 months.

Individual M3 spoke his first word at 8 months but subsequent language development was slow. At 2.5 years, he was referred for paediatric review due to developmental delay. By 2 years and 7 months he had 15 – 18 words. His parents described him as shy with good behaviour. There were no concerns with his general health, and he had been discharged by

the otolaryngology and ophthalmology teams. MRI head scan was normal. From 7 months to 2.5 years of age, he required medication for constipation.

On examination at 2 years and 10 months of age, individual M3 had a weight of 16.7 kg (+1.4 SD), height 92 cm (-0.6 SD) and OFC 52.5 cm (+0.9 SD). He had deep set eyes, up-slanting palpebral fissures, prominent dark eyelashes and eyebrows, low set ears, inverted nipples, short toes and tapering fingers. His hair, teeth and nails were normal. His social interactions were normal. By age 5 years, individual M3's parents reported that food seeking behaviour had started to become a major issue. His penile length was at the lower end of normal range. Endocrinology review is planned at puberty.

Individual M3 was recruited to the DDD study (DECIPHER 295010). However, his history of hypotonia, developmental delay and dysmorphic features, meant Börjeson-Forssman-Lehmann syndrome (BFLS) was considered as a possible diagnosis. Single gene testing found a 3 base pair deletion in *PHF6* c.1014_1016del, p.(Glu338del). The variant was maternally inherited. His mother did not have any features of BFLS.

Family A, individual M4

This male patient was the maternal cousin of individual M3. His mother was the sister of individuals M1 and M2. He was born at 40+3 weeks gestation. No problems were reported during the pregnancy or birth. His birth weight was 2920 g (-1.3 SD) and birth head circumference 34.5 cm (-0.6 SD). A neonatal hearing test identified mild sensorineural hearing loss. He had hearing aids from 8 weeks of age. Individual M4 sat independently at 6 months and walked at 23 months. He was hypotonic and required speech and language therapy. At 22 months, he had less than 10 words. An ophthalmology assessment identified a left convergent squint and long-sightedness.

At age 28 months, his weight was 11.9kg (-1.0 SD), height 85.4cm (-1.3 SD) and head circumference 48.8cm (-1.4 SD). He had prominent ears and a prominent metopic suture.

At age 5.5 years, his weight was 20.7 kg (+0.4 SD) and his height was 108cm (-1.0 SD). He was assessed as having global developmental delay.

At age 6 years 3 months, his weight was 23.2 kg (+0.6 D) and his height was 112.8 cm (-0.9 SD). He was attended a special school. At age 6 years and 10 months, his parents reported that he had generally good behaviour with occasional angry outbursts.

Individual M4's investigations included creatine kinase, thyroid function test, urinalysis, an electrocardiogram, routine karyotype, and *GJB2*/*GJB6* gene testing. The results were all normal. Sanger sequencing confirmed that he had the familial *PHF6* variant c.1014_1016del, p.(Glu338del). His facial features were in keeping with a diagnosis of BFLS.

Family B, individual M5

This male patient was born at term by normal vaginal delivery. His birth weight was 3033 g (-1.1 SD). There were no issues during the pregnancy or delivery. As a neonate, he was floppy with poor head control. His gross motor milestones were delayed. After he attained independent walking, he remained ‘clumsy’ with a tendency to fall. Significant speech and language delay was noted. He had difficulty with understanding full sentences and complex instructions.

Ophthalmology assessment identified nystagmus and hypermetropia. At age 2 years of age he had multiple partial seizures over a number of weeks. These were confirmed with EEG and have been well controlled on a single anti-epileptic medication.

On examination at 7 years 5 months of age, individual M5 was noted to have large ears with fleshy earlobes, deep set eyes with a heavy brow ridge and inverted epicanthus. His fingers were short and tapered. He also had truncal obesity and spastic diplegia. His skin, hair and genitalia were normal.

Individual M5 attends a special school due to moderate intellectual disability. His personality is described as friendly and affectionate. No behavioural issues have been noted. He is very aware of other people’s emotions.

An MRI brain scan has shown hypomyelination and some asymmetric features suggestive of an antenatal insult. Genetic investigation found a paternally inherited benign copy number variant on 15q26.3 and a maternally inherited variant of uncertain clinical significance in *MAMLD1* c.2573C>T, p.(Pro858Leu).

The patient was recruited to the DDD study (DECIPHER 294422) which identified a maternally inherited *PHF6* variant c.1014_1016del, p.(Glu338del). No other members of his are reported to have features of BFLS. No connection between family A and B has been identified.

Family C, individual M6

This male patient was born at 31 weeks gestation by emergency caesarean section due to maternal pre-eclampsia. His birth weight was 1474 g (-0.6 SD). He was admitted to the special care baby unit for 3 weeks and required CPAP. He had bilateral inguinal hernias and a ventricular septal defect. Within the first few months, he was noted to have scaphocephaly and required surgery for this and for the hernias.

Individual M6 walked at 15 months. He spoke single words until 18 months of age. There were no concerns with his vision or hearing. At age 3, he was diagnosed with epilepsy, for which he takes sodium valproate.

At age 8 years, he had poor concentration and could not read or write. He was awaiting an assessment for autistic spectrum disorder. He had poor co-ordination and struggled with fine motor skills. On examination, his growth parameters were within the normal range: weight 25th to 50th centile, height 25th centile and OFC 9th to 25th centile. He had hypertelorism with small alae nasi, bulbous nasal tip, thin upper lip with downturned lower lip and a wide

philtrum. His eyebrows were deficient laterally. He had prominent ears, tapering fingers and two frontal cowlicks. He also had a small penis and had been referred to the Paediatric Endocrinologists.

Individual M6 had an array CGH which identified a maternally inherited 624 kb 17q25.3 deletion of uncertain clinical significance. He was recruited to the DDD study (DECIPHER 280090) which reported a maternally inherited missense variant in *PHF6* c.1057G>C, p.(Asp353His). His mother had facial features similar to those reported in BFLS carrier females. However, the *PHF6* variant was classified as a variant of uncertain clinical significance.

Family D, individual M7

This male patient presented to the Clinical Genetics Service due to a history of moderate global developmental delay. He was born at term. Birth weight was 3440 g (-0.2 SD). At birth he was noted to have an umbilical hernia and bilateral single palmar creases. His walked independently at 23 months of age. His first words were at 23 months of age.

On examination, at 2 years 5 months of age, individual M7 was noted to be small. His weight was 10.4 kg (-2.4 SD), height 79.2 cm (-3.4 SD) and OFC 47.5 cm (-2.5 SD). He had unilateral ptosis, fine hair, hypotonia, pes planus and abnormal dental morphology. His facial features were consistent with a diagnosis of BFLS. His MRI brain scan was normal.

Individual M7 was recruited to the DDD study (DECIPHER 274397) which identified a *de novo* variant in *PHF6* c.1024C>T, p.(Arg342X).

Family E, individual M8

This male patient was born at 38 weeks gestation by normal vaginal delivery. His birth weight was 2636 g (-1.1 SD). His mother was born with cleft lip and palate. She had hypopituitarism and mild intellectual disability. His father had a history of epilepsy. Individual M8 required two weeks of phototherapy. He had surgery for undescended. No other neonatal issues were reported.

Individual M8 had delayed gross motor skills: he sat at 8 months, crawled at 19 months and walked at 3 years. At 2.5 years, he was referred to Clinical Genetics due to developmental delay and dysmorphic features. He had no meaningful speech and his grasp remained immature. He spoke his first word at 3.5 years and at age 5 years, was able to put two words together. He achieved pincer grasp at 4 years.

On examination at 7 years of age growth parameters were height 111.6cm (-2.0 SD), weight 25.2kg (+0.6 SD) and OFC 50.6cm (-1.9 SD). He had nystagmus, left-sided hearing impairment, and severe intellectual disability. He also had growth hormone deficiency. On examination, he had large ears with posterior helical pits, 5th finger clinodactyly, tapering fingers, short 4th toes, a high palate, tongue tie and inverted nipples. No other skin abnormalities were identified.

Investigations included renal ultrasound which identified a left pelvic kidney. Brain MRI showed delayed myelination. A metabolic screen was normal, as was routine karyotype. Array CGH identified a duplication of Yq11.223. Individual M8 was recruited to the DDD study (DECIPHER 259005) which identified a maternally inherited variant in *PHF6* c.686A>G, p.(His229Arg).

Family F, individuals M9 and M10

Individuals M9 and M10 are brothers who were previously described in detail as children [1,2].

Family G, individual M11

This male patient presented to his local Clinical Genetics service at 37 years of age. Detailed information about his birth and development were unavailable. He had a history of right-sided talipes and delayed language skills. At 2 years of age, he was not walking independently.

At 37 years of age, he had severe intellectual disability. His behaviour was described as occasionally aggressive. On examination he had long ears with large fleshy ear lobes, tapering fingers, brachydactyly of the toes, gynaecomastia, small short palpebral fissures, hypertelorism and a broad nose. Growth parameters were unavailable.

Single gene testing identified a variant in the first codon of *PHF6* c.2T>C, p.(Met1Tyr). His mother was unavailable for testing. His two healthy sisters opted for testing and had not inherited the mutation.

Family H, individual M12

This male patient was born through an IVF pregnancy. Decreased fetal movements were reported during the pregnancy. Individual M12 was born at term and was noted to have significant hypotonia. He was admitted to the NICU due to a suspected infection. He was discharged home after one week. Sitting and crawling were delayed. Walking was achieved at 24 months. Fine motor skills were poor from a young age.

At age 10 years 2 months individual M12 had mild intellectual disability and was described as having narrow interests and lacking in initiative. He was also sensitive to tactile stimuli and when playing, tended to play in parallel, usually with younger children. M12 had an “unstoppable want for food” which had improved more recently, although he remained overweight. He found it hard to fall asleep, woke during the night, and then found it hard to wake up in the morning. On examination, he had prominent supraorbital ridges, gynaecomastia, tapering fingers, short 2nd toes and large ears with fleshy helices. His weight was 58 kg (+2.7 SD) and his height was 149.7 cm (+1.7 SD). He had several, small hypopigmented macules, a few café au lait spots and acanthosis nigricans of the axillae.

His early hypotonia and motor delay led to concerns about an underlying muscle condition. EEG, EMG and muscle biopsy were all normal. Endocrine investigations were performed

due to small genitalia. Testosterone, LH and FSH were normal. Array CGH and testing for Prader-Willi Syndrome and Myotonic Dystrophy Type 1 were normal. Trio exome sequencing identified a missense variant in *PHF6* c.146C>T, p.(Ser49Leu). The variant was maternally inherited but *de novo* in the mother of individual M12.

Family I, individual F1

This female patient was born at term after a normal pregnancy with a birth weight of 3572 g (+0.4 SD). She made no respiratory effort for 5 minutes after birth and required bag and mask ventilation. She did not feed well for the first 48 hours and was late in gaining head control. She sat at 13 months and walked at 23 months. Her speech was also delayed with very limited speech at age 3 years. She had recurrent glue ear and was diagnosed with bilateral sensorineural hearing loss at 3.5 years of age. She also had bilateral blocked nasolacrimal ducts and had a tonsillectomy.

On examination at 4 years and 6 months of age individual F1 had low muscle tone and OFC was 75th to 90th centile. She had deep set eyes, long eye lashes, medially flared eyebrows with mild synophrys, a short, pinched nose with small alae nasi, a depressed nasal bridge, and slight mid-face hypoplasia. Her skin displayed patchy, streaky hyperpigmentation in the groins and inner thighs. She had tapering fingers, 5th finger clinodactyly, short 2nd toes, and 2-3 toe syndactyly. She had fine hair and slow growing hypoplastic/ dystrophic nails (particularly right 5th finger and all toes except halluces). Her teeth were noted to be small with poor enamel. Potential diagnoses considered included Rothmund-Thomson syndrome and incontenti pigmenti. Ophthalmology examination was normal. A skin biopsy from a patch of pigmented skin on her leg was normal.

Individual F1 had her first seizures at 9 months of age. Seizures tended to be nocturnal and continued despite medication. At age 6 years, her seizures were still poorly controlled with four anti-epileptic medications. At age 7 years, a change in medication led to 9-month seizure free period of being seizure free during which her behaviour improved. An MRI brain scan was reported to show blurring of the grey-white matter junction of the right frontal lobe (inferior and middle frontal gyri) extending into the right insula and possibly the right temporal lobe. The appearance was suggestive of a cortical dysplasia. The left hemisphere appeared normal.

There were no concerns about her early social development. She attended a full-time nursery and then a mainstream school with 1:1 support. However, her verbal age was significantly delayed and in mid-childhood, she was felt to be displaying autistic features. At age 10, her OFC was 53.2cm (-0.4 SD). The streaky pigmentation on her trunk and legs appeared to fade as she became older.

At 15 years of age, individual F1 had a weight of 77.1 kg (+2.3 SD) and height 167.1 cm (+0.8 SD) test. Her puberty was delayed. She had some breast development and axillary hair but minimal pubic hair. The primary amenorrhoea was managed with Estriol Gel. Ultrasound showed absent ovaries and rudimentary uterus. Endocrine investigations showed low gonadotrophins, normal prolactin, low oestradiol and undetectable progesterone. Thyroid function tests were normal. Her other investigations included a neurometabolic screen, array CGH, *IKBKG* gene testing and testing for Fragile X syndrome. The results were all normal.

Trio whole exome sequencing identified a *de novo* heterozygous variant in *PHF6* c.743G>T, p.(Gly248Val). X-inactivation studies showed complete (100:0) skewing.

Family J, individual F2

This female patient was born at term by caesarean section due to fetal distress. There were no issues reported during the pregnancy. Her birth weight was 4224 g (+1.7 SD). She had right sided talipes, a dislocated left hip and dysmorphic features. She failed a newborn hearing test. Smiling was achieved at 3 months. At 8 months she was unable to sit without support. From early in life it was thought that individual F2 might have an overgrowth disorder. At 8 months old, her weight was 8.38 kg (+0.1 SD), length 69.1 cm (+0.1 SD) and OFC 45.2 cm (+0.4 SD). She developed a large umbilical hernia and subsequent renal ultrasound scanning identified a dilated renal pelvis and megaureter. At age 2 years 4 months, she had significant cognitive and language delay. She was able to make some noises but had no recognisable speech.

At age 5 years, she communicated by Makaton and gesturing. On examination, her dysmorphic features included hypertelorism, a pointed nose, long philtrum, 5th finger clinodactyly, short toes, right foot 4-5 syndactyly, prominent nasal bridge, prominent upper lip and up-slanting palpebral fissures, a sloping forehead and fleshy ears. She had hyperextensible joints and truncal hypotonia. As a young child, she had chronic otitis media.

As individual F2 got older, her parents reported increasing hair on her back and that she snored. During childhood, her behaviour became more challenging with autistic features. She had a statement of special educational need. Eye examination identified a right-sided squint, hypermetropia and unusual pigmentary and atrophic changes affecting the choroid and retina. Her vision was unchanged between age 4 and age 15 years. Echocardiography found a small patent foramen ovale and mild mitral valve prolapse. Dental examination found hypoplastic maxilla, hypodontia with small, widely-spaced and irregularly shaped teeth, dens invaginatus and compound composite odontoma.

Individual F2 had increasing deformity of her fingers, mainly clawing of the 2nd and 5th digits. X-rays showed significant shortening of middle phalanx of both little fingers with no epiphyseal plate and a degree of shortening of the 4th and 5th metacarpals bilaterally.

At 15 years of age her weight was 74.4 kg (+2.1 SD) and height 165 cm (+0.5 SD). At 16 years of age her OFC was 56.6cm (+0.9 SD). Her puberty had progressed appropriately with normal breast development, although she had sparse body hair.

Individual F2 had small, deep-set toenails that were difficult to cut. At age 9 years a lump was removed from under her right big toe toenail. The lesion recurred and was removed again at 12 years of age. Similar swellings developed on other toes. By 16 years of age, individual F2 had had nail bed ablation of several toes. All the biopsied lesions showed similar histological features: dome shaped dermal spindle cell lesions covered by atrophic epidermis. The spindle cells were in a dense fibrotic stroma of collagen fibres and thin-walled compressed blood vessels. The cells were cytologically bland and showed no inclusion bodies (H&E and trichrome stains) in contrast to infantile digital fibromas which they resembled clinically. Very occasional mitotic figures were seen. On immunostaining there was considerable expression of smooth muscle actin and some patchy staining of desmin.

Calponin, CD34, S100, factor 8 or factor 13a were negative. Initially the lesions were described as similar to early keloid scar or dermal leiomyoma. However, the appearance and behaviour were noted to be unusual for a leiomyoma. Instead, a diagnosis of digital infantile fibromatosis was proposed. The combination of non-inclusion body digital fibromas, blaschkoid pigmentation and digital anomalies in individual F2 led to consideration of a diagnosis of Terminal Osseous Dysplasia with Pigmentary Defects (TOPD). However, *FLNA* gene testing was normal.

Individual F2's other skin features included macular streaky brown pigmentation in her axillae, flanks, inguinal folds and thighs. She developed extensive hypertrophic scars due to acne. The scars mainly affect her chest and shoulders, but isolated lesions extend down to her buttocks. She also had areas of follicular keratosis and follicular atrophoderma.

Individual F2 developed swelling of her right knee and varicose veins. She is now 26 years old and continues to have recurrent bluish purple bulbous swellings growing from toes of both feet. The lesions cause pain, chronically ulcerate, and limit mobility.

Individual F2's genetic investigations included a skin biopsy of a pigmented skin patch at 4 years of age which found low-level mosaicism for Turner syndrome (45,X in 11 out of 150 cells examined). Routine karyotype of blood was normal (50 cells examined). Karyotype of skin fibroblasts from a chest keloid confirmed low level mosaicism for Turner syndrome (45,X in 19 out of 528 cells examined [3.3%]). Single gene tests of *NSD1*, *EP300* and *CREBBP* were normal. However, clinical array CGH identified a heterozygous 206 kb deletion involving exons 6 to 10 of *PHF6* and the adjacent *HRPT1* gene (arr[GRCh37]Xq26.2q26.3(133546221_133751899)x1). The result is entered on the DECIPHER database (DECIPHER 340129). X-inactivation studies (on blood) showed complete (100:0) skewing.

Family K, individual F3

This patient was born by normal vaginal delivery at 40+5 weeks gestation with a birth weight of 3480 g (+0.2 SD). She spent five days on the special care baby unit due to low blood sugars and low temperature. Parental concerns about plagiocephaly eventually led to referral to the Paediatrics and Clinical Genetics. She was noted to have developmental delay and dysmorphic features. Individual F3 sat at age 1, stood with support at 21 months and walked at 4.5 years. She was also slow to develop fine motor skills and displayed significant delay in speech and language. At age 12 years, individual F3 uses Makaton but had better understanding compared to her expressive communication ability.

At age 10 years, individual F3's family began noticed progressive thinning of her calves. On examination she had significant calf muscle wasting with preserved thigh and upper limb muscle bulk. Muscle tone was increased, and she had eversion contractures with limited dorsiflexion and brisk knee jerks. Despite progressive wasting and a positive Gower's sign, her balance and walking distance on the flat improved. Electromyography showed markedly abnormal motor findings limited to the lower limbs. Sensory studies were normal. Her upper limbs were unaffected.

Individual F3 had recurrent middle ear infections and required grommets. She also had a squint, refractive error and an umbilical hernia. At 11 years of age, she was unable to read or

write and had not achieved toilet training and was a poor sleeper. Individual F3 enjoys food but is liable to aspiration with thin fluids. There have been no reports of seizures. She has also been diagnosed with bilateral physeal bars which presented with intense pain and for which she has had surgery.

At age 11 years of age, individual F3's weight was 28.95 kg (-1.2 SD), height 125.8 cm (-2.7 SD) and OFC 52.7 cm (-1.1 SD). On examination, her features include prominent brow ridge, hypertelorism, thick dark eyebrows with synophrys, down-turned corners of the mouth, thick upper lip, short upturned nose, high anterior hairline with temporal sparseness. Her teeth were peg-shaped. Her skin displayed widespread patchy hyper and hypopigmentation. Both axillae and groins have acanthosis nigricans and she had a hirsute back. Her left ear had a large earlobe and the right was low set. Her hands and feet were cool with large big toes and laterally deviated toes. Her fingers were tapering with second to fifth finger brachydactyly (5th more marked) and fifth finger clinodactyly. Individual F3 had scoliosis. An MRI scan showed an S-shaped scoliosis with congenitally missing thoracic/lumbar vertebrae.

Individual F3's genetic investigations included a routine karyotype, *IKBKG* gene testing and a neuropathy gene panel. The results were all normal. Array CGH identified an 11 kb deletion at Xq26.2 This involved exon 4 and 5 of *PHF6* and was confirmed with quantitative PCR. Parental testing confirmed it was *de novo*.

Family L, individual F4

This patient was born at term by normal vaginal delivery with a birth weight of 2600 g (-0.5 SD). She was referred to Clinical Genetics at 18 months of age due to delayed development. She achieved sitting at 17 months and walking at 25 months. Her cry was described as 'cat-like', prompting laryngeal investigations, which were normal. She displayed photophobia and a subsequent eye examination identified dysplastic optic discs with a mottled epithelial appearance. She also has hypermetropia. She had bilateral conductive hearing loss secondary to otitis media but this resolved.

At 12.5 years, individual F4 was able to talk in sentences and had severe intellectual disability. She displayed autistic traits, hand wringing and could be inappropriately affectionate. She also had anxiety and a sleep cycle disorder. Due to hyperphagia, her weight was 60.6 kg (+1.8 SD) compared to her height which was 153.4 cm (+0.1 SD). Her last OFC measurement at age 6.5 years was 51.7cm (-0.6 SD).

On examination, individual F4 had dysmorphic features including brachycephaly, a short neck, thick arched eyebrows, long eyelashes, prominent supra-orbital ridges, a high nasal root, long fleshy philtrum, thin upper lip vermillion and small teeth. Her ears were long with large anteverted ear lobes. Her hair was thick and she had 5th finger clinodactyly, small nails, and broad feet. Her toes were short, particularly the 4th and 5th which also display partial syndactyly. At age 3 years of age, linear hyperpigmentation of the neck, axillae and groin was noted. She also had extensive blashkoid hyperpigmentation on the abdomen. Examination of her genitalia identified hypoplastic labia minora and no vaginal orifice. An ultrasound of the pelvis showed a small uterus and ovaries.

Investigations included an MRI brain scan, echocardiogram, an array CGH and *ARID1B* gene testing. The results were all normal. She was recruited to the DDD study (DECIPHER

258725) which identified a *de novo* heterozygous variant in *PHF6* c.955C>T, p.(Arg319*). This mutation had previously been identified in a female with similar features including small teeth, syndactyly and hypoplastic clitoris and labia majora [3].

Family M, individual F5

This female patient was born at 37 weeks gestation by ventouse delivery with a birth weight of 3100 g (+0.8 SD). A raised nuchal translucency was identified on antenatal scans but invasive testing was declined. Postnatally, she was hydropic and had an umbilical hernia, positional talipes, ptosis and streaky skin hyperpigmentation. She required a chest drain for spontaneous pneumothorax.

Gross motor milestones were delayed. Head control was achieved after 6 months, sitting at 9 months and walking at 23 months. Ophthalmology review identified mild myopia and poor depth perception. She had grommet insertion and fluctuating conductive hearing loss. Her speech was a little unclear and her comprehension was better than her expressive language.

Initially she was om mainstream schooling with support, but this was changed to a special school at 9 years of age. She had mild to moderate intellectual disability. She disliked any change to her routine, could be anxious at times and had some distinctive mannerisms.

At night, she required CPAP for obstructive sleep apnoea. She was also prone to recurrent urinary tract infections. No seizures were reported. Surgery included several adenoidectomies, an operation for a left dislocated hip, and correction of a divergent squint. At age 15, she was amenorrhoeic. She had sparse, slow-growing scalp hair and swirly hyperpigmentation on left hip and thigh

Her genetic investigations included skin chromosome analysis, Prader-Willi syndrome methylation analysis and *IKBKG* gene testing. An MRI brain scan showed mild ventricular dilatation, a relatively enlarged cerebellum and increased signal in the periventricular white matter. Echocardiography, renal ultrasound scan and neurometabolic blood tests were normal. Individual F5 was recruited to the DDD study (DECIPHER 262468) which identified a 342 kb *de novo* Xq26.2-26.3 microdeletion which included all of the *PHF6* gene. (arr[GRCh37]Xq26.2q26.3(133497871_133839836)x1)

Family N, individual F6

This female patient was born at term with a birth weight of 3402 g (+0.0 SD). She displayed features of global developmental delay from a young age. Her acquisition of gross motor, fine motor and speech and language skills was delayed. Although individual F6 had no formal diagnosis of epilepsy, she was noted to have staring episodes. She required a tonsillectomy and adenoidectomy due to obstructive sleep apnoea. Other long-term health issues included chronic sinusitis, constipation and oropharyngeal-phase dysphagia.

At age 10 years, individual F6 had a diagnosis of autism. Her non-verbal IQ was 62 and her verbal IQ was 40. Hyperphagia was a significant concern, and her weight was at the 99th centile with other growth parameters at the top end of the normal range (height 96th and head

circumference 95th centile). She had distinctive features including narrow palpebral fissures, deep-set eyes, prominent supraorbital ridges, hooded eyelids, a short narrow nose with an upturned tip, a sloping forehead, frontal bossing and a receding lateral hairline. She also had 5th finger clinodactyly with large, narrow ears and widely spaced, irregular teeth. When making a fist, nodules on the phalanges were visible. Apart from a superficial haemangioma on the nape of her neck, she had no other skin features of note.

An ultrasound identified asymmetric kidneys. Her MRI brain scan was unremarkable. Blood tests highlighted a raised triglyceride level. X-rays of her hands showed hypoplastic middle and distal phalanges of the 5th finger. Array CGH reported an 87 kb deletion at 1q44 involving part of the *SMYD3* gene. The deletion was classified as a variant of uncertain significance. Exome sequencing identified a heterozygous missense variant in *PHF6* c.732G>C, p.(Leu244Phe). The variant was not identified in her mother; her father was not available for testing. The exome sequencing also identified a heterozygous missense variant in the *MFN2* gene (NM_014874.4): c.899G>A, p.(Arg300His). The *MFN2* variant is present in gnomAD (3 alleles) and Clinvar (Variation ID: 1195317). The *MFN2* variant is currently classified as a variant of uncertain clinical significance.

Family O, individual F7

This female patient was born at 41+1 weeks with a birth weight of 3400 g (+0.0 SD). Apart from neonatal jaundice, no other issues were reported in early life. She sat at 11 months and walked at 27 months. Due to concerns about her development, she was referred to the Clinical Genetics Service at age 2.5 years. Her speech was delayed and by age 9, she was speaking in sentences and using Makaton. She had a moderate level of intellectual disability and attended a special school.

On examination, at 15 years of age, her weight was 91.3 kg (+3.2 SD), height 158.8 cm (-0.5 SD) and OFC 60 cm (+3.6 SD). She had tapering fingers, broad feet, a sandal gap, small deep set toes nails, fleshy ear lobes and streaky pigmentation on her back. She had coarse facial features with puffiness around the eyes, a short nose and long philtrum. She had primary amenorrhoea but with some breast and hair development.

Individual F7's brain MRI scan identified hypoplasia of the corpus callosum and cavum septum pellucidum. A metabolic screen was normal. Genetic investigations had included routine karyotype, array CGH, *FRAX* gene testing, and methylation studies for Prader-Willi syndrome. The results were all normal. Individual F7 was recruited to the DDD study (DECIPHER 304952) which reported a *de novo* heterozygous missense mutation in *PHF6* c.719A>G, p.(Tyr240Cys).

Family P, individual F8

This female patient was born following a complex pregnancy. Her mother had lupus and there was premature rupture of membranes at 23 weeks gestation resulting in oligohydramnios. There were antenatal bleeds at 29 and 31 weeks. She was born at 35 weeks gestation due to a partial placental rupture. Her birth weight was 1990 g (-1.1 SD). Feeding issues were immediate and global developmental delay was suspected from an early age. She sat at 18 months and walked at 2.5 years. Fine motor skills were also poor. OFC at 2 years 2

months was 47.5cm (-1.5 SD). At age 3 years, she was diagnosed with a Wilms tumour that was treated by nephrectomy and chemotherapy.

Individual F8's acquisition of speech was slow. At age 4 years, she had 25 words and at age 7 years, she spoke in short sentences. There were no concerns about her social skills. She displayed various stereotypies including winking, moving her mouth and making noises. Her intellectual deficit was in the moderate range. At 6 years 5 months her weight was 26.4 kg (+1.3 SD) and height 115 cm (-0.6 SD). On examination, she had a pinched nose with narrow nasal passages, a broad forehead and poor hair growth. On her right foot, she had a mild right hallux valgus and the 2nd and 3rd toes were short and syndactylous. Her fingers were short. Her ears and genitalia were normal. She has a tendency towards dental caries.

Ophthalmology assessment identified an alternating divergent squint with normal acuity. She has a history of otitis media, glue ear and mild obstructive sleep apnoea. Chest X-ray identified 11 pairs of ribs. 7-dehydrocholesterol level was normal. Genetic testing included karyotype (blood and skin) and array CGH. The results were normal. She was recruited into the DDD study (DECIPHER 286430) which identified of a *de novo* heterozygous variant in *PHF6* c.297T>A, p.(Cys99*).

References

1. Carter MT, Picketts DJ, Hunter AG, Graham GE. Further clinical delineation of the Börjeson-Forssman-Lehmann syndrome in patients with PHF6 mutations. Am J Med Genet A. 2009;149A: 246–50.

2. Vallée D, Chevrier E, Graham GE, et al. A novel PHF6 mutation results in enhanced exon skipping and mild Börjeson-Forssman-Lehmann syndrome. J Med Genet. 2004;41: 778–83.

3. Zweier C, Kraus C, Brueton L, et al. A new face of Borjeson-Forssman-Lehmann syndrome? De novo mutations in PHF6 in seven females with a distinct phenotype. J Med Genet. 2013;50: 838–47.
